# MicroRNA-34 family expression in bovine gametes and preimplantation embryos

**DOI:** 10.1186/1477-7827-12-85

**Published:** 2014-09-02

**Authors:** Allison Tscherner, Graham Gilchrist, Natasha Smith, Patrick Blondin, Daniel Gillis, Jonathan LaMarre

**Affiliations:** Department of Biomedical Sciences, Ontario Veterinary College, University of Guelph, 50 Stone Road East, Guelph, Canada; L’Alliance Boviteq, 19320 Rang Grand St François Ouest, Saint-Hyacinthe, Canada; Department of Mathematics and Statistics, College of Physical and Engineering Science, University of Guelph, 50 Stone Road East, Guelph, Canada

**Keywords:** Gametogenesis, MicroRNA, Biomarkers

## Abstract

**Background:**

Oocyte fertilization and successful embryo implantation are key events marking the onset of pregnancy. In sexually reproducing organisms, embryogenesis begins with the fusion of two haploid gametes, each of which has undergone progressive stages of maturation. In the final stages of oocyte maturation, minimal transcriptional activity is present and regulation of gene expression occurs primarily at the post-transcriptional level. MicroRNAs (miRNA) are potent effectors of post-transcriptional gene silencing and recent evidence demonstrates that the miR-34 family of miRNA are involved in both spermatogenesis and early events of embryogenesis.

**Methods:**

The profile of miR-34 miRNAs has not been characterized in gametes or embryos of *Bos taurus*. We therefore used quantitative reverse transcription PCR (qRT-PCR) to examine this family of miRNAs: miR-34a, -34b and -34c as well as their precursors in bovine gametes and *in vitro* produced embryos. Oocytes were aspirated from antral follicles of bovine ovaries, and sperm cells were isolated from semen samples of 10 bulls with unknown fertility status. Immature and *in vitro* matured oocytes, as well as cleaved embryos, were collected in pools. Gametes, embryos and ovarian and testis tissues were purified for RNA.

**Results:**

All members of the miR-34 family are present in bovine spermatozoa, while only miR-34a and -34c are present in oocytes and cleaved (2-cell) embryos. Mir-34c demonstrates variation among different bulls and is consistently expressed throughout oocyte maturation and in the embryo. The primary transcript of the miR-34b/c bicistron is abundant in the testes and present in ovarian tissue but undetectable in oocytes and in mature spermatozoa.

**Conclusions:**

The combination of these findings suggest that miR-34 miRNAs may be required in developing bovine gametes of both sexes, as well as in embryos, and that primary miR-34b/c processing takes place before the completion of gametogenesis. Individual variation in sperm miR-34 family abundance may offer potential as a biomarker of male bovine fertility.

## Background

Terminal maturation is the final series of nuclear and cytoplasmic events that bring a haploid gamete to a state where it has the capability to contribute to a fertilization event and establish an embryo. The maturing oocyte undergoes dynamic morphological and nuclear rearrangements as it approaches ovulation, progressively decreasing its transcriptional activities [1] together with the condensation of genetic material. At this time, the oocyte contains a repertoire of RNAs that will guide it through the early stages of embryogenesis until the resumption of transcription occurs at the time of embryonic genome activation [2]. Critical to this onset of zygotic transcription and further embryonic development is the destruction of a number of maternally deposited transcripts [3].

The formation of mature male gametes is equally complex. Diploid spermatogonia are formed from germline stem cells that undergo mitotic divisions and differentiation to produce primary spermatocytes [4]. Through meiosis, the chromosome number of these spermatocytes is reduced resulting in haploid spermatids. During the final steps of spermiogenesis, round haploid spermatids take on the distinctive morphological characteristics of mature spermatozoa [5]. The transformation of round spermatids into spermatozoa involves nuclear elongation and a high degree of DNA compaction in the sperm head [6]. This reorganization results in the arrest of transcription during mid-spermiogenesis, however, a number of transcripts persist in ejaculated spermatozoa [7].

Paternal contributions to the zygote and its development are increasingly recognized as important element of successful fertilization. Sperm are now known to provide proteins and RNAs that are critical to subsequent development. Some mRNAs present in sperm and early embryos are absent from non-fertilized oocytes, suggesting a unique role for these RNAs in post-fertilization events [8].

MicroRNAs (miRNAs) have, over the past several years, emerged as potent regulators of gene expression that are widely expressed in biological systems. These small non-coding RNAs are highly conserved among eukaryotes and a growing body of evidence supports major roles in developmental, homeostatic and pathological processes. MiRNAs typically act by binding to complementary sequences in the 3′ untranslated region of target mRNAs and inhibiting gene expression by accelerated transcript decay or translational suppression through interactions with the multiprotein RNA-induced silencing complex (RISC) [9]. Their roles in numerous biological pathways have highlighted the complexity of post-transcriptional gene regulation. While many miRNAs are ubiquitously expressed, tissue-specific miRNA expression is common, suggesting unique requirements in different tissues and specific functional roles. The roles of miRNAs are likely to be particularly important in tissues where transcription is limited, resulting in an environment where post-transcriptional regulatory mechanisms predominate.

MicroRNAs are transcribed by RNA polymerase II [10] as primary “pri-miRNA,” and are processed to “pre-miRNA” by a multi subunit microprocessor complex containing the RNaseIII enzyme Drosha [11] and subsequently into mature miRNA by the bidentate RNaseIII ribonuclease Dicer [12]. Mammalian miRNAs have an average decay rate in somatic cells of approximately 5 days and are up to ten times more stable than mRNAs [13]. Their known roles as potent silencers, and their intrinsic stability makes them strong candidates for the regulation of gene expression in gametes and embryos.

Considerable numbers of miRNAs are present in oocytes, many of which exhibit dynamic expression profiles. A subset of miRNAs demonstrate increased abundance through oocyte maturation and embryo development. Conversely, some miRNAs that are abundant in the immature oocyte become depleted throughout maturation, while the abundance of others remains relatively stable [14]. The dynamic expression profiles of miRNAs throughout early embryogenesis strongly implicate them in the timely regulation of embryonic gene expression.

The transcriptome of spermatozoa includes both long (tRNA, rRNA and mRNA) and small RNAs including miRNA, endo-siRNA, piRNA [15, 16], and recently described short noncoding sperm RNAs (spRNA) [17]. In addition to their potential regulatory roles in early embryonic development, miRNAs are present in the testis and profiling has revealed populations of miRNA that are either preferentially or exclusively expressed in this tissue [18]. These miRNAs may be required for the completion of spermatogenesis, and the loss of miRNA processing machinery in testes results in severe disruptions to this process [19]. A survey of human sperm has uncovered a unique set of primary miRNA transcripts present in sperm cells that are essentially absent from the testis, and are not observable in their short, mature form in the sperm. It has been suggested that these pri-miRNA precursors may be delivered to the oocyte upon fertilization, and are cleaved into their mature form only in the embryo, where they begin targeting mRNAs [20]. The small RNA profiles of sperm may ultimately represent a useful parameter that is correlated with male fertility, as high versus low fertility-status bulls have been shown to exhibit different miRNA signatures [21].

The miR-34 family consists of three miRNAs (a,b,c) which contain identical seed regions and show variable tissue expression. Gametes of several species have been shown to contain miR-34 family members that appear important in both gametogenesis [22] and early embryo developmental events [23, 24]. In somatic cells, miR-34 is an integral component of the p53 network, impeding cell cycle progression and proliferation by silencing oncogenic targets [25]. The abundance of the miR-34 family is transcriptionally regulated by p53 [26]. MiR-34b and -34c suppress proliferation and colony formation in neoplastic epithelial ovarian cells, and the binding of p53 to a conserved site on the miR-34b/c promoter increases the expression of these miRNA in ovarian surface epithelium cells, an effect lost after the conditional inactivation of p53 [27]. The roles of miR-34 in reproduction are incompletely understood and may be p53 independent, as p53 deficient mice testes show only a slight difference in miR-34c compared to their normal counterparts [22]. Furthermore, miR-34 availability in the gametes may be more dependent on pri-miR-34 processing, as p53 regulates miR-34 at a transcriptional level.

While miR-34a appears widely expressed, miR-34c is detected in only a small subset of tissues, particularly the gonads [28]. In the testes of mice it is found primarily in germ cells where expression increases concomitantly with the differentiation of pachytene spermatocytes into round spermatids where the highest levels are present [22]. MiR-34b and miR-34c are found in zona pellucida-bound sperm, suggesting an association with fertilization capacity [23]. MiR-34c is also abundant in human sperm and present in the testis, but absent from the human ovary [15]. Small RNA profiling of human seminal plasma in normozoospermic versus vasectomized donors has revealed that miR-34c is one of a select group of miRNA that is markedly reduced or undetectable in vasectomized individuals [29], suggesting that miR-34c present in semen is either preferentially or exclusively derived from the testis/epididymis and that its absence may be a diagnostic of impaired fertility. The simultaneous inactivation of miR-34b/c and its functionally related family member miR-449 - which contains an identical seed sequence, result in male sterility in mice due to severely altered epididymis as well as low sperm counts and deformed sperm with minimal motility [30].

The miR-34 profile in female gametes is highly variable between species. MiR-34b and -34c are found in mouse zygotes and appear to be required for the first zygotic cleavage event, the expression level is comparable to that of sperm, and it is not detected in unfertilized oocytes [23]. In contrast, a single miR-34 orthologue (to miR-34a) is present in the oocytes of *Drosophila melanogaster.* The miR-34 family is present in oocytes of zebrafish and is maternally inherited in embryos of these two species [24]. To date, the miR-34 family has not been examined for its role in bovine reproduction.

Based on the expression and characteristics of the miR-34 family members in the gametes of other species, we designed the present study to characterize this family of miRNAs in male and female bovine gametes in order to establish the dynamics of their processing, their potential as candidates of paternally contributed zygotic RNAs.

## Methods

### Sperm preparation

Semen samples were collected from 9 Holstein bulls and 1 Jersey bull owned by and housed at EastGen (Guelph, Ontario, Canada), and arbitrarily assigned Animal Identification numbers from 1–10. Frozen semen was thawed in water at 37°C and overlayed onto a discontinuous 45% and 90% density gradient of Percoll (Sigma-Aldrich, St. Louis, MO) diluted in HEPES-buffered Tyrode’s albumin-lactate-pyruvate (TALP) medium (Caisson Labs, North Logan, UT) with 1.97 mM CaCl_2_, 0.39 mM MgCl_2_, 25.6 mM Na lactate and 25 mM NaHCO_3_. Sperm cells were purified from cryoprotectant and somatic cell contamination by centrifugation at ambient temperature for 30 minutes at 700 *g*. After the careful removal of the Percoll solution, sperm pellets were washed in 5 mL HEPES-TALP and collected by centrifugation at 700 *g* for 5 minutes. Purified, motile sperm were diluted 50 fold in nuclease-free water for counting by haemocytometer, and 3 pools of 4x10^6^ sperm per animal were flash frozen in liquid nitrogen.

### Oocyte collection and in vitro fertilization

Oocyte collection and *in vitro* embryo production was performed as described previously [31]. Bovine ovaries were obtained from a local abattoir (Cargill, Guelph, Ontario, Canada) and transported at 35°C. Within 2 hours of ovary collection, cumulus-oocyte complexes (COC) were aspirated from follicles greater than 6 mm diameter using vacuum aspiration. Collected complexes were placed into 1 M HEPES-buffered Nutrient Mixture F-10 Ham (Sigma-Aldrich) collection media supplemented with 2% steer serum, Hepalene, penicillin and streptomycin.

COC were washed twice in HEPES-buffered TCM-199 maturation medium (Caisson Labs) supplemented with sodium pyruvate (Sigma-Aldrich), 0.2 mol/L L-glutamine (Sigma-Aldrich) and 0.6% penicillin-streptomycin (Invitrogen, Canada).

Immature oocytes were denuded of associated cumulus cells in 2 mg/mL Hyaluronidase from *Streptomyces hyalurolyticus* (Sigma-Aldrich), washed in phosphate-buffered saline plus 0.01% poly vinyl alcohol and collected into pools of 40 and immediately flash frozen in liquid nitrogen.

Oocytes for maturation were placed in groups of 15 under mineral oil in 80 μL drops of maturation medium containing 0.5 μg/mL FSH, 1 μg/mL LH and 1 μg/mL estradiol (NIH, USA) in a humidified atmosphere at 38.5°C and 5% CO_2._ After 22 hours, COCs were either denuded and collected or fertilized.

In preparation for fertilization, *in vitro* matured oocytes were washed in HEPES-TALP (Caisson Labs) and fertilized in groups of 15 under oil in 80 μL drops of TL-Fert (Caisson Labs) fertilization medium supplemented with 20 μg/mL heparin sodium salt (Sigma-Aldrich), and 0.96 μg/mL albumin from bovine serum (BSA) (Sigma-Aldrich). It is unknown whether miR-34 family miRNAs present in bovine sperm are transferred to the oocyte upon fertilization. To eliminate the potential for variation in miR-34 levels due to paternal contribution, all oocytes used for embryo production in this study were fertilized with cryopreserved bovine sperm from one bull that was not used in any analyses of miR-34 variation between bulls. Frozen semen was washed in modified HEPES-TALP (Caisson Labs) and prepared by conventional swim-up technique. At 18 hours post fertilization, presumptive zygotes were denuded by vortexing and cultured in groups of 25 in 30 μL drops of Synthetic Oviduct Fluid (Caisson Labs) supplemented with 0.96 μg/mL BSA, 88.6 μg/mL sodium pyruvate, 2% non-essential amino acids (Sigma-Aldrich), 1% essential amino acids (Sigma-Aldrich), 0.5% gentamicin, and 2% serum. Cleaved (2-cell) embryos were collected at 36 hours post fertilization.

### Tissue collection

Testicular tissue was obtained from University of Guelph Abattoir (Guelph, Ontario, Canada) immediately after slaughter and was flash frozen on-site. Ovarian cortices were dissected on ice from bovine ovaries collected and transported from the abattoir (Cargill) at 35°C, and were immediately flash frozen in liquid nitrogen.

### RNA extraction and cDNA synthesis

Total RNA including small RNAs was extracted from gametes and tissues using miRNeasy Micro kit (Qiagen, Mississauga, Canada) according to the manufacturer’s protocol, and with the inclusion of DNase digestion performed on-column with the RNase-free DNase Set (Qiagen). RNA was extracted from 3 different pools of 4x10^6^ sperm from each bull, from 3 pools of 40 oocytes or embryos at each of the stages studied, and from testes and ovarian cortices of 3 male and female cattle. RNA concentration and quality was measured by Nanodrop 2000c (Thermo Scientific, Wilmington, DE).

Extracts were reverse transcribed with either qScript microRNA cDNA Synthesis Kit (Quanta BioSciences, Inc., Gaithersburg, MD) or qScript cDNA SuperMix (Quanta Biosciences).

### Quantitative PCR

Quantitative RT-PCR analysis was performed on samples after reverse transcription using a CFX96 Touch Real-Time PCR Detection System (BioRad Laboratories, Inc., Hercules, CA). Pri-miRNAs were amplified using SsoFast EvaGreen SuperMix (BioRad). Mature miRNAs were amplified with PerfeCTa SYBR Green SuperMix, a specific miRNA Assay Primer, and PerfeCTa Universal PCR primer (Quanta BioSciences). All miRNA Assay Primers used in this study were purchased commercially (Quanta Biosciences), and are based on human miRNA and snRNA sequences. Expression was validated in bovine. The 5′ arm (5p) primers were selected for bta-miR-34a, -34b and -34c by analysis of deep sequencing reads of 5′ and 3′ arm expression in bovine tissues available in miRBase Registry [32]. Primer efficiencies were determined by standard curve. Relative miRNA expression was calculated by efficiency-corrected ΔΔCt method, normalized to the endogenous control snRNA U6 which has been shown previously to be an internal control stable in embryo culture systems [33]. The amount of cDNA template used per reaction represents the equivalent RNA of one oocyte/embryo, or 3 ng sperm or tissue RNA. Primary miR-34b/c primers were designed using Primer 3 software [34] against the genomic region flanking the miR-34b/c stem-loops in the UMD3.1 assembly of the bovine genome [35]. The following oligonucleotides were used to amplify a segment of pri-bta-miR-34b/c; Forward: 5′-TTGGCGAGGAGGATTGGAA-3′ and Reverse: 5′-TGTGTAGCTTTCCCCAGCGA-3′. PCR products were purified from 2% agarose gels with QIAquick Gel Extraction Kit (Qiagen) and sequenced to verify primer specificity. The reference genes 18S ribosomal RNA: Forward: 5′-CGGCTACCACATCCTATGAA-3′, Reverse: 5′-TGGAGCTGGAATTACCGCGG-3′, and YWHAZ (validated for its expression in bovine embryos [36]): Forward: 5′-GCATCCCACAGACTATTTCC-3′, Reverse: 5′-GCAAAGACAATGACAGACCA-3′ were used as endogenous controls for RT-PCR.

### Statistical analysis

Differences in miRNA abundance between tissues and stages of oocyte development were analyzed with the Kruskal-Wallis test and Dunn’s *post hoc* test for multiple comparisons using GraphPad Prism 6. Differences with a P-value <0.05 were considered significant.

## Results and discussion

### miR-34 family is expressed in bovine sperm and testis

**Figure 1:**
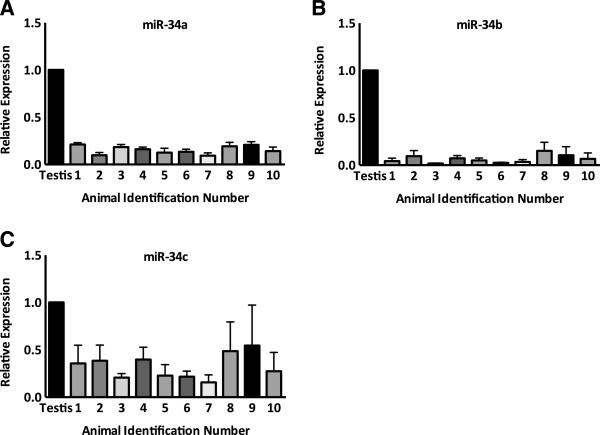
**MiR-34 family abundance in bovine spermatozoa. A)** miR-34a, **B)** miR-34b and **C)** miR-34c expression determined by qRT-PCR on pools of 4x10^6^ sperm isolated from 10 individuals (n = 3), normalized to the endogenous control snRNA U6. Values presented as a relative fold-change against a calibrator of testis RNA. Error bars represent SEM.

MiR-34 family miRNA levels were measured in sperm isolates purified from ejaculate samples of 10 individual bulls. Three pools of 4x10^6^ sperm were prepared from cryopreserved sperm isolated from each bull, and miRNA extraction/qPCR quantification from the triplicate pools of each individual were carried out separately in order to assess the potential for technical variability within the experimental design. Sectioned testis tissue, which contains a large number of differentiating spermatocytes, was found to abundantly express miR-34a, -34b and -34c. For this reason, a sample of testis cDNA was used as a calibrator against which all sperm samples were compared. Multiple samples of testis cDNA were tested and the calibrator selected was chosen based on its similar Ct value to the sperm samples for the endogenous control snRNA U6.MiR-34a, -34b and -34c were detected in the sperm from all individuals tested (Figure 1A-C) at a median threshold cycle (Ct) among all animals of 30.9, 35.9 and 27.5, respectively. Mean mir-34a expression values in each bull ranged from 9-21% of the expression level in the testis calibrator, while mean miR-34b ranged from 2-15%, and miR-34c ranged from 16-55% of the calibrator expression value. Of the three genes in this family of miRNAs, miR-34c was the most readily detected in all animals tested, and showed the widest range of expression between individuals.

This blinded study was carried out with no prior knowledge on the underlying structure of miR-34c distribution, or its relationship with bovine fertility. It is possible that the observed variation may arise from differential transcription, proportion of motile, high-quality sperm in semen samples, or underlying genetics. The three members of the miR-34 family all map to single locations in the UMD3.1 genome assembly of the domestic cow. This most current release of the bovine genome includes 12,251 structural variants, however a search of this assembly did not reveal any annotated copy number variants (CNVs) surrounding the miR-34a, -34b or -34c loci. For comparative purposes the GRCh37.p13 release of the human genome was obtained from Ensembl [37] and revealed annotated gains and losses of copies of the miR-34a allele on human chromosome 1 and at the miR-34b and -34c loci on human chromosome 11. Copy number variation is often manifested phenotypically and is an important source of genetic diversity. CNVs have been detected among different cattle breeds [38] and between individuals of the same breed [39]. Variation in the copy number of miR-34 family genes represents a possible source of variable miRNA expression between individual bulls, and quantification of these alleles compared to a reference genome is a promising direction for further studies.

### miR-34a and -34c, but not miR-34b, are expressed in bovine oocytes and during in vitro embryo development

**Figure 2:**
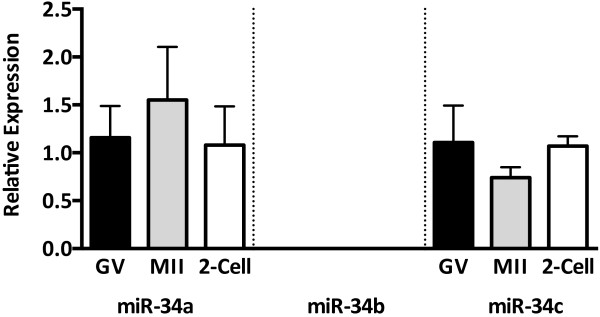
**MiR-34 family abundance in oocytes and embryos.** MicroRNA expression determined by qRT-PCR on pools of 40 oocytes at germinal vesicle (GV) stage, metaphase II (MII), and 2-cell embryos (n = 3), normalized to U6. Values presented as a relative fold-change against GV oocytes. Error bars represent SEM.

Members of the miR-34 family are transferred from sperm to oocyte in the mouse and are essential for subsequent cleavage events [23]. To determine miR-34 family expression in female gametes and investigate the possibility that the presence of miR-34c in the zygote is of paternal origin, we quantified the abundance of miR-34a, -34b and -34c in immature/germinal vesicle (GV) oocytes, mature/metaphase II (MII) oocytes and cleaved (2-cell) *in vitro* produced embryos. MiR-34a and -34c were stably expressed from germinal vesicle-stage oocyte to the 2-cell embryo, while miR-34b was not detected (Figure 2). Dynamic changes in the abundance of many miRNAs occur throughout oocyte meiotic maturation, suggesting active regulation of specific target transcripts in this final period of oocyte development. Since miR-34a and -34c persist from the immature oocyte to the cleaved embryo, it is possible that they are functionally active during this period, or that they may be required in later stages of pre-implantation embryo development if they are comparably present during *in vivo* development. Knockdown of miR-34 in zebrafish oocytes results in embryonic dysregulation of a number of known mammalian miR-34 target transcripts including the anti-apoptotic factor Bcl-2, as well as Notch and its ligand Delta-like 1, resulting in defective brain development [24]. The relationship between miR-34c and Bcl-2 is relevant to both spermatogenesis and embryogenesis. In mouse testes, miR-34c inhibition increases Bcl-2 expression and results in apoptosis-resistant germ cells. These effects may be mediated by the Bcl-2 transcription factor ATF-1; also a miR-34c target demonstrating increased expression as a result of miR-34c inhibition [40]. Bcl-2 itself is a direct miR-34c target with an anti-proliferative function and miR-34c inhibition in mouse zygotes results in Bcl-2 protein overexpression and an inability to complete the first zygotic cleavage [23]. The presence of miR-34c in the bovine oocyte and embryo suggests that similar regulation occurs, but that it does not likely depend on a paternal contribution of this miRNA.

### The bicistronic primary transcript of miR-34b/c is present in reproductive tissues but not in mature gametes

**Figure 3:**
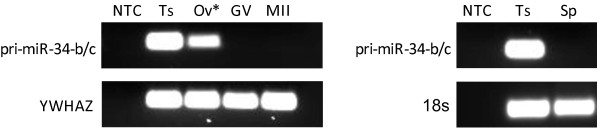
**Pri-miR-34b/c expression in gametes and reproductive tissues.** RT-PCR of testis (Ts), ovarian cortex (Ov), germinal vesicle (GV) or metaphase II (MII) oocytes, and sperm (Sp), (n = 3). YWHAZ and 18s used as reference genes. *Pri-miR-34b/c detected in ovarian cortex from 1 of 3 individuals tested.

In the bovine, the miR-34 family is transcribed from two intergenic genomic locations; miR-34a is found on the forward strand of *Bos taurus* chromosome 16 (chr16: 45,197,390-45,197,496), and miR-34b/c are co-transcribed from a single transcript on chromosome 15 (chr15: 22,134,725-22,134,808 and chr15: 22,135,426022,135,502) [32, 35]. Our finding that miR-34c but not -34b is found in bovine oocytes and embryos led us to investigate the presence of the miR-34b/c primary transcript in the ovary, testis, and gametes. An RT-PCR assay was designed to amplify the primary transcript that contains miR-34b and -34c, which was found in testis and in 1 of 3 ovarian tissue samples tested but not in mature sperm or oocytes (Figure 3). The absence of primary miR-34b/c in mature gametes suggests that the processing of this precursor takes place earlier in gametogenesis, and underscores the need for functionally active miR-34c in the oocyte and both miR-34b and -34c in sperm.

The findings in the present study differ from miR-34 processing dynamics in the mouse, where pre-miR-34c is present in mature sperm [23]. This demonstrates species-specificity not only of miRNA expression profiles in gametes and embryogenesis, but also unique differences in the stages at which miRNA undergo processing into their functional form.

Because the miR-34c found in the oocyte must originally have been derived from its primary transcript, the observed challenges in detecting this transcript in the ovary was surprising. Ovarian cortices were dissected in an attempt to capture not only the somatic cells within the ovary, but also very small follicles that would not have been retrieved by follicular aspiration. It is possible that pri-miR-34b/c is uniquely transcribed in primordial oocytes and is not detectable in a sample that has not been enriched for these small, infrequent cells. It is important to note that the findings described here have been generated using *in vitro* matured embryos. This is clearly relevant to the wider issue of reproduction, as the utilization of bovine *in vitro* embryo production systems is essential for the genetic improvement of livestock globally. *In vitro* embryo development also represents a strong and well-recognized model of oocyte maturation and early embryogenesis *in vivo.* However, differences in the pattern of gene expression between *in vitro* and *in vivo* embryo development have been recognized [41], suggesting that differences may be present in miR-34 biology. This will require confirmation with embryos derived *in vivo* in future studies.

The presence of primary miR-34b/c in the testis (containing many developing spermatocytes) and absence from mature spermatozoa parallels the pattern found in the ovary. Pri-miR-34b/c processing therefore is likely associated with early bovine gametogenesis, and the stability of miR-34b and miR-34c are unequal in the oocyte, as miR-34b is not detectable by the time the follicle has reached ~6 mm (the time of follicular aspiration and collection), while miR-34c is present in its functional form and remains through to the first cleavage event. Many miRNAs are found in evolutionarily conserved clusters, and their transcription is initiated by a single promoter. As such, polycistronic miRNA are often up- or down-regulated jointly in response to changing cellular conditions [42]. Though it is common, equal abundance of co-transcribed miRNA is not universal. Inconsistent expression of miRNA derived from a single cluster has been previously documented, and data suggests that the miR-34b/c transcript may be an example of an unequally expressed miRNA cluster [43]. Furthermore, miR-34b/c itself has been recently found to undergo alternative tissue-specific splicing that depends on competition between spliceosome and miRNA-processing machineries [44]. It is possible that a similar competitive process may account for the differences we observe in mature miR-34b and miR-34c, which are derived from the same primary transcript.

### The miR-34 family is variably expressed in bovine reproductive tissues

**Figure 4:**
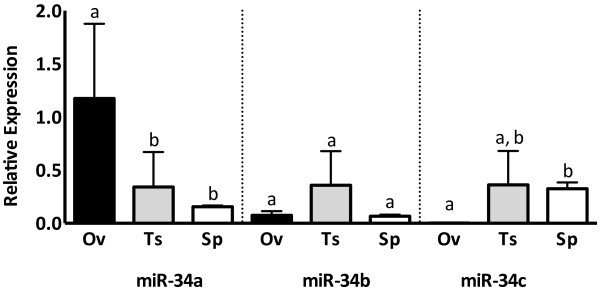
**MiR-34 family abundance in sperm and reproductive tissues.** MicroRNA expression determined by qRT-PCR on ovary (Ov), testis (Ts) tissues and sperm (Sp), normalized to U6. Values reported are the mean expression in ovary and testis tissues isolated from 3 individual cattle, and the mean expression of sperm from 10 bulls. Data presented as a relative fold-change against testis RNA. Error bars represent SEM, letters represent groups whose means differ significantly (P < 0.05) for each gene.

The levels of miR-34 family miRNAs vary between male and female reproductive tissues and sperm (Figure 4). While miR-34a is most abundant in the ovary, miR-34c is scarcely detectable in sections of ovarian tissue. Conversely, miR-34a is not highly expressed in the testis or in sperm, while miR-34c is abundant in testis and sperm. Interestingly, while miR-34c expression in ovarian tissue is low, miR-34c is abundant in oocytes and embryos, suggesting that miR-34c is enriched in the oocyte compared to the somatic cells in the ovary.

Of the miR-34 family, miR-34c was found to be the most interesting candidate in both male and female gametes with respect to individual variability and possible enrichment compared to the surrounding tissue. The presence of miR-34c in mature spermatozoa isolated from ejaculates suggests that the role of miR-34c in bovine reproduction may not only involve spermatogenesis, but it may also have potential roles in fertilization and subsequent embryo development. Human studies have demonstrated that miR-34c is highly abundant in ejaculates of donors with proven fertility [15], and this potential putative function of miR-34c in cattle is further emphasized by the observed variation of miR-34c among individuals. With the unknown fertility status of our samples, miR-34c presents itself as a prospective biomolecule for further study as a marker for male reproductive competence. The scope of this study was to perform the first characterization of miR-34 family miRNA in bovine gametes and associated reproductive tissues. Through a large cohort study, these small RNA candidates could be examined for correlation with other parameters of sperm quality and with known fertility status.

Routine evaluations to assess the quality of bull semen are a largely qualitative physical inspection of sperm morphology, motility and concentration [45]. These parameters often fail to predict fertilization, and studies have shown that molecular defects correlated with fertilization failure can exist in morphologically normal sperm [46]. Our results suggest that miR-34c may have the potential to be used as a non-invasive and quantifiable measure to assess reproductive competency in bovine, and may be used in conjunction with current predictors of sperm quality.

## Abbreviations

FSH: Follicle-stimulating hormone
GV: Germinal vesicle
LH: Luteinizing hormone
MII: Metaphase II
qRT-PCR: Quantitative reverse transcription PCR.
